# The plasma membrane as a mechanochemical transducer

**DOI:** 10.1098/rstb.2018.0221

**Published:** 2019-07-01

**Authors:** Anabel-Lise Le Roux, Xarxa Quiroga, Nikhil Walani, Marino Arroyo, Pere Roca-Cusachs

**Affiliations:** 1 Institute for Bioengineering of Catalonia (IBEC), The Barcelona Institute for Science and Technology (BIST), Barcelona 08028, Spain; 2 LaCàN, Universitat Politècnica de Catalunya-BarcelonaTech, Spain; 3 Department of Biomedical Sciences, Universitat de Barcelona, Barcelona 08036, Spain

**Keywords:** plasma membrane, mechanotransduction, membrane tension, mechanosensor

## Abstract

Cells are constantly submitted to external mechanical stresses, which they must withstand and respond to. By forming a physical boundary between cells and their environment that is also a biochemical platform, the plasma membrane (PM) is a key interface mediating both cellular response to mechanical stimuli, and subsequent biochemical responses. Here, we review the role of the PM as a mechanosensing structure. We first analyse how the PM responds to mechanical stresses, and then discuss how this mechanical response triggers downstream biochemical responses. The molecular players involved in PM mechanochemical transduction include sensors of membrane unfolding, membrane tension, membrane curvature or membrane domain rearrangement. These sensors trigger signalling cascades fundamental both in healthy scenarios and in diseases such as cancer, which cells harness to maintain integrity, keep or restore homeostasis and adapt to their external environment.

This article is part of a discussion meeting issue ‘Forces in cancer: interdisciplinary approaches in tumour mechanobiology’.

## 1. Introduction

By forming a physical boundary permitting the segregation of specific chemical reactions, the self-association of amphiphilic lipid molecules played an important role in the origin of life. Accordingly, the plasma membrane (PM) of prokaryotes and eukaryotes constitutes a fundamental border between the cell and its environment, and tightly regulates the exchanges between the inside and outside of the cell. Its physical state and integrity are crucial for cell survival, and a major function of the PM is to preserve its integrity and enable changes in cell shape. These changes occur in response not only to cell processes such as division, migration or spreading, but also to the constant external mechanical forces present in physiological scenarios. Mechanical stimuli destabilize cellular homeostasis and are strongly associated with cancer [1,2], and cells need to respond to either maintain their integrity or trigger appropriate responses.

In this context, the PM constitutes a crucial interface, since mechanical forces will result in a change of its state. Accordingly, extensive work (which we cite here non-exhaustively) has addressed how membrane tension interplays with the actin cytoskeleton (CSK) to regulate cell shape [3–5], motility [6–9] and polarity [10,11]. Correspondingly, many reviews discuss the feedback between membrane mechanical properties, CSK organization and cell dynamics [12–19]. Whereas in this review we will not analyse this feedback in detail, we will address a related and equally important topic: how the PM can harness mechanically induced changes in its state to itself act as a mechanosensor. We will first detail the different types of external mechanical stimuli that can be applied to the PM, and its subsequent mechanical response. Then, we will review how this PM response triggers specific molecular mechanosensing events, mediated by diverse mechanosensory molecules that share a common principle: they are sensitive to a mechanical state of the PM, or to a change of this state. Accordingly, they transduce the external mechanical input transmitted from the PM into a biochemical response. To decouple the effect of each particular signal, we will not consider inputs composed of multiple mechanical signals (such as those usually present in three-dimensional geometries). Therefore, we limit the review to two-dimensional *in vitro* systems and examine the acute response of mammalian cells to a single mechanical input.

## 2. Effect of external mechanical stimuli on the plasma membrane

The highly complex PM delimits the cell, and is in permanent contact with its surroundings. As such, it constitutes a crucial interface, since interactions with or alterations from the external environment will result in a change of its mechanical state. Whereas all living cells (eukaryotic or prokaryotic) receive a large diversity of mechanical inputs, here we will focus on the PM of animal cells. Examples of such cells in a mechanically active environment include alveolar epithelial cells in lungs, which cyclically stretch and relax, or skin cells, which also experience transient stretch, but without a cyclic rhythm. Cells in the intestinal track withstand transmural pressure, shear flow and cyclic strains [20], and smooth muscle cells in the bladder [21] or in ocular cells [22] are exposed to hydrostatic pressure. Vascular endothelial cells, circulating cells such as red blood cells, and cells from the immune system are exposed to shear flows [23,24]. Among a plethora of other cells, circulating cells (RBC)s passing in the kidney medullae [25], cells in the renal medullae [26] and cells exposed to the environment undergo important osmotic changes, which will stretch or compress cells. Topography is another external crucial mechanical cue, as many adherent cells must physically adapt to a topographically heterogeneous substrate such as the extracellular matrix (ECM) [27]. From a mechanical perspective, these stimuli can be grouped as tensile stresses (as applied by cell stretching and hypo-osmotic treatments), compressive stresses (as applied by cell compression and hyper-osmotic treatments) and shear stresses (as applied by shear flows acting on adherent cells) [28]. In this section, we will focus on experiments mainly applying only one of these stimuli, and we will not analyse their combined effects (as could occur for instance in cells flowing through narrow constrictions) [29]. As such, we will not consider three-dimensional geometries as they would lead to complex mechanical inputs.

### (a) Tensile stresses

**Figure 1.**
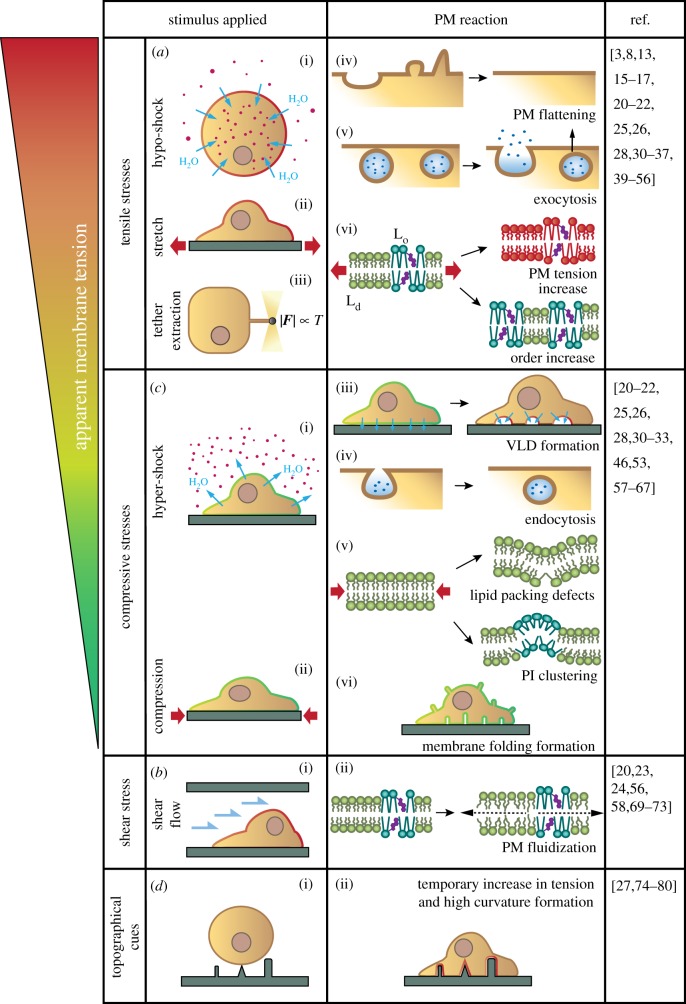
PM response to applied mechanical stimuli. (*a*) Tensile stresses are applied experimentally by tether pulling, hypotonic shocks and cell stretching. In response, PM folds flatten and exocytosis increases, buffering the increase in tension. Once lipid reserves have been used, PM tension and order increase. (*b*) Compressive stresses are applied experimentally through hyper-osmotic shocks and stretch release. In response, PM folds of different shapes and sizes form (vacuole-like dilations (VLDs), tubes), endocytosis increases, lipid packing defects appear in highly curved areas and phosphoinositide (PI) clusters form. (*c*) Shear stress application results in increased PM fluidity, in both L_o_ and L_d_ phases. (*d*) Upon encountering topographical cues, cells adapt their PM to substrate architecture, likely triggering a temporary increase in PM tension.

Tensile stresses on the PM are commonly applied through hypotonic treatments [30–32], both to adhering and suspended cells (figure 1*a*(i)). Cells respond by swelling, thereby expanding their volume. Such osmotic treatments raise the issue of potential chemical responses triggered in parallel to the mechanical response [33,34]. Alternatively, cells adhered to flexible membranes can be stretched uniaxially or equibiaxially [31,35] (figure 1*a*(ii)), thereby regulating PM area more directly. In both types of experiment, there is an increase in PM apparent tension, a measure containing the PM tension and the tension induced by adhesion to the underlying CSK, to which the PM is strongly attached (see further details in box 2). Indeed, application of 40–70% osmotic treatments increases apparent tension in neuron cells [32], fibroblasts [3] and human leukaemia cells (HL-60) [36], and application of a 40% uniaxial stretch increases apparent tension in human leukaemia cells [36]. In these assays, tension most likely increases both in the PM and in the underlying cytoskeletal actin cortex. The CSK is in fact thought to have mechano protective effects on the poorly extensible PM, which resists only a 3–5% area expansion before lysis, or rupture [8,28]. The CSK prevents tension-induced PM rupture by absorbing part of the applied stress [33,37]. Consistently, apparent PM tensions in unstressed cells range from 0.03 to 0.3 mN m^−1^ [16,38], far from the estimated rupture tension of a bilayer, 3–10 mN m^−1^ [8,13,38,39].

Upon stress application, however, cells have multiple ways of supplying lipids to the PM to buffer tension increases. First, highly abundant PM folds (see box 1) flatten upon tensile stress application [15,30,31] (figure 1*a*(iv)). Caveolae are an important type of such folds [40], exerting a PM mechanoprotective role directly [41] (rather than through caveolae endocytosis). Second, exocytosis contributes to PM area expansion [35,42–45] by adding lipids to the bilayer. Exocytosis occurs in response to PM tension increase [38] (figure 1*a*(v)). Interestingly, simple membrane mechanics could drive this process, as stretching a supported lipid bilayer leads to passive absorption of liposomes sitting on top of it [46]. Similarly, endocytosis arrest has been associated with increased PM tension, as observed for instance during osmotic swelling of rat basophilic leukaemia (RBL) cells [47]. Here again, tension may facilitate molecularly driven exocytosis [38,39].

Box 1. **Mechanical and molecular features of the plasma membrane**

The cellular PM has evolved to become an extremely complex entity, bearing an asymmetric bilayer considered as a two-dimensional fluid formed by glycerophospholipids, sphingolipids, cholesterol and carbohydrates, as well as high amounts of transmembrane or peripheral proteins. A key PM parameter is its fluidity (also referred as its reciprocal viscosity, which is the internal property of a fluid that offers resistance to flow [23]). Fluidity is commonly related to high molecular mobility in the bilayer, which enables lateral diffusion of the embedded molecules [73]. Diffusion in the PM is slower than in a pure lipid bilayer [179] because of its lateral organization, especially through the presence of peripheral and transmembrane proteins [180], its shape and its attachment to the cytoskeleton (CSK). The complex composition of the PM results in lateral liquid–liquid phase separation of the bilayer in liquid-disordered (L_d_) and liquid-ordered (L_o_) domains, with reduced diffusion in the latter. L_o_ domains in the PM (also called lipid rafts) are enriched in cholesterol, sphingolipids and transmembrane or anchored proteins, display a higher lipid packing and dynamically assemble and disassemble at a fast rate in domains of different sizes [181]. Of note, controversies remain about the organization and dynamics of these domains [182].

Another fundamental PM parameter is its topography. The PM has a spontaneous curvature conferred by its composition and asymmetry and is not a smooth bilayer [183]. It contains actively maintained folds in the form of micro- and nano-structures such as ruffles, microvilli or caveolae [13]. Among them, caveolae are small PM invaginations (20–100 nm) shaped by caveolin and cavin proteins [102,184,185]. Caveolae are enriched in glycosphingolipids and cholesterol but are apparently devoid of transmembrane proteins [186]. These folds are actively maintained through mechanochemical feedbacks which have been the object of many studies [80,132,187]. The PM is also strongly coupled to the CSK through different biochemical links. ERM proteins (ezrin, radixin and moesin proteins) [188] mediate PM attachment to the cortex, and actin filaments also attach to caveolae [184], lipid rafts [189] and recently described asters (actin-based PM nanoclusters) [181]. Such PM–CSK interaction may locally impair diffusion and organize the PM as a ‘fence and picket’ bilayer [190]. The PM constantly undergoes fusion (exocytic) and fission (endocytic) events through a variety of pathways [191], ensuring protein and lipid turnover as well as chemical communication with the outside environment. Endocytosis decreases PM area while exocytosis increases it.

Overall, the cell capacity to expand its area under tensile stress will thus depend not only on the nature and the magnitude of the stretch, but also on the PM lipid reserves in the form of folds and endomembranes [48]. For instance, the abundance of caveolae is cell-type-dependent, and they are highly abundant specifically in smooth muscle cells (highly exposed to stretch), and in endothelial cells (highly exposed to shear flows) [49]. Moreover, cells have very different resting tensions depending on their type [17,33] and state [3,48] and may even display heterogeneous apparent tension distribution within the PM upon application of local stimuli [50]. Such differences may explain why the cellular response to osmotic treatment is strongly cell-type-dependent: in a range of cell types, PM area expansion prevented tension increases upon hypotonic treatments of 50%, but not of 98% [43]. However, in mouse lung endothelial (MLEC) cells, a 50% hypotonic treatment did not lead to an increase in tension unless caveolar proteins were knocked down [40]. Additionally, the physiological relevance of large osmotic treatments in most physiological situations was questioned in a recent study [51], and for instance a value of 20% (measured to be insufficient to affect tension) was found to be closer to physiological values in endothelial cells. In any case, the magnitude of osmotic treatment or stretch needed to increase PM tension after cells have depleted their lipid reserves, and its relationship to the relevant physiology of each cell type, remain an open question.

At the molecular level, an increase in PM tension (and thereby membrane area) is predicted to decrease its thickness, to minimize PM volume and the exposure of hydrophobic tails to water [28]. This could directly affect transmembrane protein conformation, decrease lipid packing and facilitate diffusion in the lipid bilayer. This was predicted in 1,2-dioleoyl-*sn*-glycero-3-phosphocholine (DOPC) bilayers [52] and observed upon application of an osmotic treatment in synthetic giant unilamellar vesicles (GUVs) made of DOPC [53]. In GUVs of complex lipid composition, bilayer tension triggered phase separation and appearance of liquid-ordered (L_o_) domains in the PM (see box 1) [53,54], which is in opposition to some theoretical predictions [55] (figure 1*a*(vi)). Increased PM order was also observed upon uniaxial stretch of vascular endothelial cells [56], coupled with a slower diffusion as assessed by FRAP (fluorescence recovery after photobleaching) measurement. Similarly, increased PM order occurred upon application of a hypoosmotic treatment in HeLa or MDCK cells [53]. Both effects (phase separation and lipid unpacking) would likely affect lipid raft organization and dynamics (see box 1). However, they would, respectively, increase or decrease packing and diffusion in the bilayer, and therefore the overall effect of PM tension remains unclear.

### (b) Compressive stresses

When stretch is released, or if a hyperosmotic treatment is applied, a decrease in apparent tension has been consistently measured [32,57,58] (figure 1*b*(i,ii)). Compression also leads to PM bending [28]. In the case of hyperosmotic treatments (or restoration of medium tonicity after an isotonic treatment), dome-shaped micrometre-sized invaginations termed vacuole-like dilations (VLDs) form passively [31–33]. VLDs are only observed at the basal substrate-bound side of the PM (figure 1*b*(iii)), and we demonstrated that they were due to expulsion from cells of water which was not absorbed by hydrophobic substrates such as glass coverslips [31]. If cells are seeded on porous substrates, water can flow through and VLDs are not observed. Upon stretch release, passive PM folds also form, both in DOPC-supported lipid bilayers [46,59] and in the PM of several cell types [31] (figure 1*b*(vi)). These folds can be dot-shaped sub-micrometre structures termed reservoirs, or longer tubes, depending on the magnitude of de-stretch. Such structures locally deform the PM, thereby accommodating the lipid excess caused by compression. Similarly, caveolae that had been unfolded by tensile stress re-form upon stress release [60]. Interestingly, VLDs can also form upon stretch release on poroelastic substrates, owing to water flow out of the substrate [31,61]. Thus, the PM can deform during compression, either by accommodating excess PM in small folds, or by generating VLDs to accommodate water effluxes. Unlike in the case of stretch, adaptation to osmotic changes does not seem to require the formation of PM structures to accommodate excess PM area, likely because, particularly in flat cells, changes in cell volume induced by osmotic treatments can be accommodated with very small changes in PM area. Consistently, another study reported that hypertonic treatments affected cell shape and volume but not PM topology [30]. In all cases and regardless of their nature, reservoirs, tubes and VLDs are subsequently actively re-absorbed in the PM after formation [31]. Endocytosis also intervenes as an additional mechanism for handling the extra lipids in the PM [62] (figure 1*b*(iv)).

Analogously to tensile stresses, compressive stresses also lead to lipid reorganization, owing to both decreased tension and formation of highly curved structures. Experiments in GUVs showed formation of lipid packing defects in highly curved structures [63,64] in both L_o_ and L_d_ phases [65], which may facilitate the insertion of hydrophobic molecules (figure 1*b*(v)). In addition, high bilayer curvature can lead to lipid sorting or changes in the lipid phases. Indeed, simulations revealed correlation between lipid clustering and lipid bilayer curvature [66]. Experimentally, pulling tubes from GUVs led to the enrichment of unsaturated compared with saturated lipids close to phase separation [67], short chain lipids have a preference for highly curved bilayers *in vitro* [63] and stretch release in yeast led to the formation of invaginations enriched in phosphoinositides (PIs) [58] (figure 1*b*(v)). Finally, a decrease in the PM tension through hyperosmotic treatment has been associated with a decrease in L_o_ areas in HeLa cells [53], which would be expected to facilitate diffusion in the bilayer.

### (c) Shear stresses

Different studies have consistently found a rapid increase in PM disorder upon shear flow application (figure 1*c*(i)), either by using Laurdan imaging (a membrane fluorescent dye sensitive to local membrane packing [56]), a molecular rotor probe [68] or an FRET-based molecular sensor [69] (figure 1*c*(ii)). Although shear flows exert both a tensile stress from the hydrostatic pressure and a shear stress on the PM [24,70], the effect was specifically attributed to shear stress in one of the studies [56]. In this study, the increased disorder was coupled to increased diffusion in both GUVs and cells. This fluidization occurs to both L_d_ and L_o_ domains (although at different time scales [71]), and in caveolae [72]. How shear stress leads to those effects is intriguing, but potential mechanisms include an increase in PM resistance to shear flow caused by L_o_ domains [73], or effects in membrane tension. Indeed, shear traction on the cell surface induced by shear flow may modify membrane tension and its uniformity, as reported in a recent study [69]. However, increased apparent tension has been associated with increased (rather than decreased) order, as discussed previously in this article.

### (d) Topographical cues

The topography surrounding cells also constitutes a mechanical stimulus, in that the cellular PM that is at the interface will be forced to adapt to the shape of the substrate (figure 1*d*(i)). *In vivo*, cells are often in contact with the ECM, a very heterogeneous material presenting changes in topography that shape the PM [27]. To mimic rough or confined topographies, cells have been seeded on micropatterned substrates of a variety of shapes, confining cell adhesion to restricted areas [74–76]. As expected, cells adapt their volume and area to the patterns. Nanoneedles [76], nanocones [77] or nanopillars [78] 50–500 nm in diameter have also been engineered on a substrate before cells were seeded on top. Electron microscopy images confirmed that the PM wrapped around the pillars, adopting a highly curved configuration [79] (figure 1*d*(ii)). Therefore, external topography deforms the PM, and probably also induces a temporary increase in tension [80].

## 3. Plasma membrane mechanical state sensors

When mechanical stimuli affect PM shape and tension, the cell responds to restore its homeostasis [33]. Fast responses occur to temporarily accommodate the new mechanical state of the PM and prevent cell lysis, while adaptative responses occur if the mechanical stimulus is repeated or maintained [81–83]. The signalling cascades triggered by the PM response rely on mechanotransduction events, in which specific mechanosensing molecules sense mechanically induced changes in the PM and trigger a biochemical response. Unravelling the nature and function of these molecules is highly relevant, since they are the first sensors in subsequent complex signalling cascades that define cell response. In this section, we will focus on the players relevant at short time scales, in response to the mechanical stimuli described above. Long-term cellular adaptation of cells to cyclic or permanent external mechanical stimuli (often abnormal and leading to disease states) goes beyond the scope of this review and we refer the reader to recent reviews on the topic [20,81].

### (a) Sensors of tensile and shear stresses

#### (i) Protein conformational changes: response to plasma membrane tension

**Figure 2.**
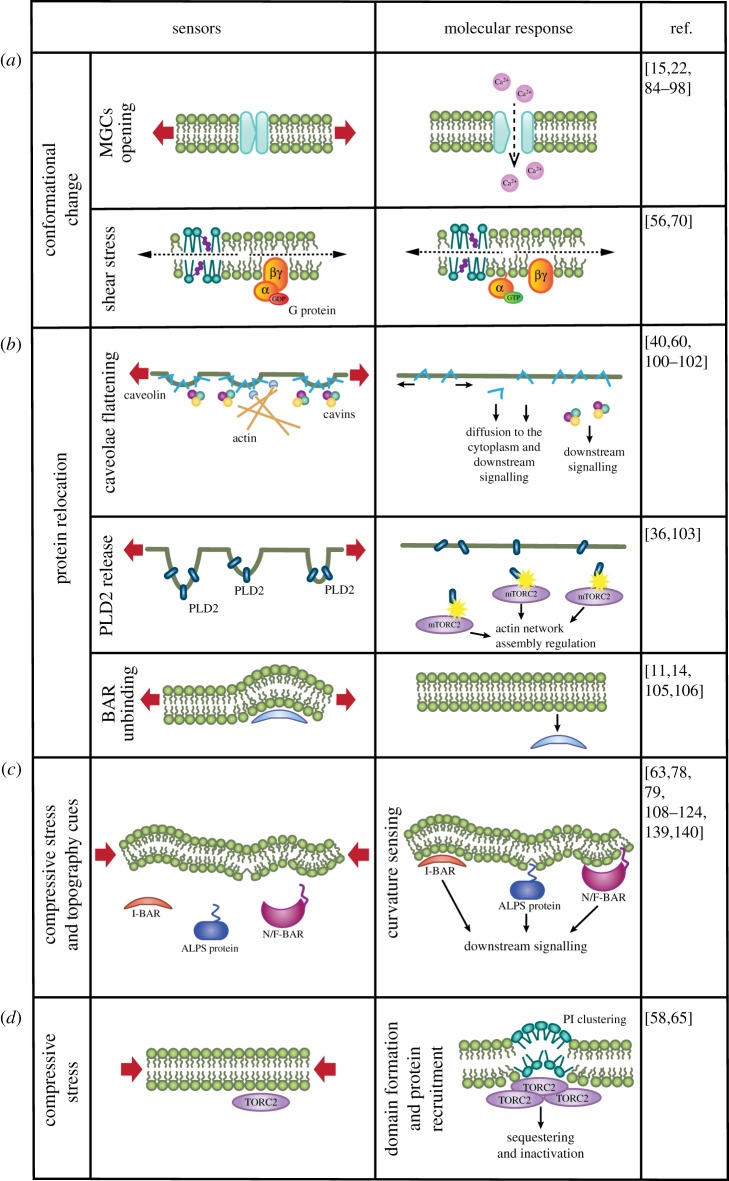
Molecular sensors of PM mechanical state. (*a*) Mechanically induced conformational changes include MGC opening upon stretch (thereby enabling ion transport) and G protein dimerization under shear flow. (*b*) Mechanically induced protein relocation events. (top) With increased PM tension, caveolae flatten and its proteic components relocate by freely diffusing through the PM and/or the cytosol. These molecular players can subsequently activate different signalling pathways. (middle) PLD2 proteins are sequestered at PM invaginations. When they unfold, PLD2 is released and activates its partner mTORC2, which subsequently regulates actin network assembly. (bottom) BAR proteins respond to fold flattening by unbinding from the PM and diffusing into the cytoplasm. (*c*) Compressive stresses and topographical cues result in the formation of different types of PM curvature that can be recognized by BAR proteins through their positively charged BAR domain. Proteins containing ALPS motifs can also sense curvature by inserting their amphipathic helix in curved PM areas, where lipid packing defects are more abundant. (*d*) Clustering of PIs due to compressive stresses leads to TORC2 sequestering and inhibition of its activity.

Mechanically gated channels (MGCs) are clear players in PM mechanotransduction [84–87]. MGCs are integral membrane proteins that undergo a conformational change in response to an area expansion of the PM (figure 2*a*). They are activated by an increase in tension due to PM stretch or suction (usually assessed through micropipette aspiration in electrophysiology assays), osmotic treatments and flow-induced shear stress [84,88,89]. In the case of shear stress, it is unclear whether the MGC responds to the shear stress itself, or to the hydrostatic pressure stretching the cellular PM. The first, bacterial channels (large conductance mechanosensitive ion channel, McsL) to be discovered have been extensively studied. These channels are believed to undergo a conformational change triggered by increased PM tension and related PM area expansion [90] (‘force from lipid’ concept [91,92]) but also possibly by the induced changes in PM thickness [93]. MscL channels are believed to open slightly before the PM reaches the rupture tension under area expansion [15,22], although this assumption may not be generalizable to the very different eukaryotic membranes. Eukaryotic PM channels include TRP (transient receptor potential) channels, potassium channels and Piezo channels [84,92,94], which have recently raised a lot of interest [95,95,96]. Their activation may be due generally to changes in PM tension, but also changes in curvature [87], and the tensions needed to activate them may not need to be as close to the rupture tension as previously believed [86,88,97]. Importantly, PM tensions include CSK contributions (see box 2), and therefore tensions needed to activate MGCs will be higher in PM regions attached to the CSK than in detached regions (blebs) or in proteo-liposomes lacking CSK [95,98]. Interestingly, whereas the important factor in PMs under stretch is generally considered to be bilayer expansion, changes in bilayer thickness may induce conformational changes by introducing a hydrophobic mismatch [28], and even clustering effects [99]. This may lead to a broader range of transmembrane proteins sensitive to PM tensile stress.

Box 2. **Plasma membrane tension**

The tension in a lipid bilayer membrane is defined as the force per unit length acting on a cross-section of a membrane [15,17,39]. Thus, PM tension is the in-plane tension, set by the nature of the lipids forming the bilayer and influenced by external forces acting on the PM of a cell. PM tension arises from hydrostatic or osmotic pressures from the cytosol, the forces exerted by the CSK and adhesion forces if the cells adheres to a substrate or other cells [15,17,39]. Whereas cell compression and micropipette aspiration can be used to assess cell tension [14], the most accurate technique involves pulling PM tethers (figure 1*a*(iii)) through atomic force microscopy, optical tweezers or magnetic tweezers [14,17], where tension is inferred from the resistance force exerted by the tether. As extensively explained [14,17,19], the tension measured with this set-up is an apparent tension containing the PM tension and the tension induced by adhesion to the underlying CSK, to which the PM is strongly attached. Tether experiments do not decouple them, except in measurements performed on PM areas detached from the CSK (blebs) [50]. Comparative measurements in or out of blebs have determined that the CSK often has a higher contribution to the apparent tension than the PM, pointing out the essential role of CSK–PM coupling [17]. Furthermore, because of the very slow equilibration of PM tension at cellular scales when coupled to the CSK, tether measurements in such a situation are a local reporter of a possibly heterogeneous tension distribution [50]. Thus, unravelling the effects of external mechanical stimuli on the PM, decoupled from those on the CSK, is an important challenge, which may be addressable through novel molecular fluorescence sensors of PM tension [53,69]. Interestingly, the cell CSK generates mechanical constraints on the PM similar to those arising from external physical forces, giving rise to comparable mechanochemical responses at the PM, with interesting implications in division, motility or spreading [3,8,14].

#### (ii) Protein conformational changes: response to fluidity

Shear stress in the PM has been linked to G protein activation, by triggering the required conformational change. Application of a shear flow resulting in increased PM fluidity seems to allow higher G protein rotational mobility, which facilitates the GDP to GTP exchange and subsequent activation [70] (figure 2*a*). Shear stress also induced phosphorylation of VEGFRs (vascular endothelial growth factor receptors) but the actual mechanism still needs to be unravelled [56].

#### (iii) Protein relocation: caveolae

Upon increased PM tension, caveolae disassemble and flatten, reducing and thereby buffering PM tension changes [40]. A primary consequence of caveolae flattening is to prevent an increase in PM tension upon stress. In terms of inducing downstream responses, PM tension buffering by caveolae could prevent MGC activation, exerting thus an indirect mechanotransduction role [100]. Beside this, caveolae disassembly facilitates the diffusion of some of their proteins (figure 2*b*). Indeed, upon application of hypo-osmotic treatment inducing a 20% swelling, an increase in non-caveolar CAV1 (caveolin-1) and a higher population of freely diffusing CAV1 have been measured in MLEC cells [40]. Subjecting myoblasts to a single fast stretch (20% uniaxial) released the binding of CAV3 (caveolin-3) to SRC (proto-oncogene tyrosine-protein kinase SRC), enhancing SRC activation [60]. In addition to caveolin release from caveolae to the bulk PM, cavin-1 (caveolae-associated protein 1) can also be released into the cytoplasm [40], and could subsequently interact with signalling effectors. Another protein, EHD2 (EH-domain-containing protein 2), has been recently shown to be released as a consequence of caveolae unfolding, and subsequently translocated to the nucleus [101]. Lipid trafficking also seems to be modified by caveolae disassembly. Interestingly, a decrease in sphingolipid packing was also measured upon caveolae flattening [60], resulting in an accelerated turnover of glycosphingolipid, which might insert more easily into the unpacked flattened caveolae. Lastly, caveolae are actively refolded upon stretch release [40] (in an ATP- and actin-dependent manner) but this active mechanism has not been elucidated in detail. However, it may involve a sensing role of a BAR (Bin/amphiphysin/Rvs) protein domain (see discussion on BAR-domain proteins below), since PACSIN 2 (protein kinase C and casein kinase substrate in neurons protein 2), which contains a BAR domain, may participate in caveolae biogenesis [102]. PACSIN 2 could thereby be an initial sensor of bilayer tension decrease, as well as a linker to actin through its SH3 (Src homology 3) domain [102].

#### (iv) Protein relocation: unfolding of other invaginations

Upon mechanical stretch (micropipette aspiration of a PM patch at 5 kPa), a redistribution of Slm (phosphatidylinositol 4,5-bisphosphate-binding protein Slm) proteins between distinct PM domains has been observed in eisosomes (folded structures) from yeast [103]. This led to activation of TORC2 (target of rapamicin kinase complex 2) and sphingolipid metabolism regulation. A similar mechanism was described recently in neutrophils [36]. Application of a tensile stress (from either hypo-osmotic treatment or a 40% radial stretch) possibly unfolded PM invaginations leading to PLD2 (phospholipase D2) release (figure 2*b*), subsequently activating mTORC2 (mammalian TORC2) and limiting actin network assembly. Another example is that of the unfolding of PM ruffles, where MARCKS (myristoylated alanine-rich C-kinase substrate) proteins localize and supposedly capture PIs. Ruffle unfolding may relocate MARCKS proteins and consequently release PIs in the PM [104]. Finally, unbinding of BAR proteins (extensively discussed in the next section) may occur upon flattening of PM invaginations [11,14,105,106] (figure 2*b*). If accompanied by an increase in PM tension, theoretical predictions suggest that BAR protein oligomerization would become unfavourable [107]. All these phenomena may potentially be enhanced by phase separation associated with increased PM tension, leading to relocation of embedded or anchored proteins.

## 4. Sensors of compressive stress and topography

### (a) Curvature sensing: Bin/amphiphysin/Rvs proteins

PM deformations generated upon compression, or because of external topography, can lead to the formation of extremely curved structures that can be detected by curvature-sensing proteins (CSPs; figure 2*c*). Among them, BAR-domain proteins are particularly relevant. Unravelling the crystal structure of AMPH (amphiphysin) [108] as a domain forming a banana-shaped arrangement upon dimerization, the so-called N-BAR domain, shed light on the way how such domains would act as scaffolds on top of curved lipid bilayers. Thereafter, N-BAR domains have been described as having a high intrinsic curvature, capable of sensing and inducing PM curvature. The family of BAR-domain-bearing proteins expanded to include different intrinsic curvatures, notably when crystal structures of F-BAR (Fes/CIP4 homology-BAR) domains (with a shallower degree of curvature than N-BAR domains) [109], and subsequently I-BAR (inverse-BAR) domains [110] (with an inverted curvature relative to N-BAR domains) followed. These domains bind the acidic PM with the positive charges of the BAR domain facing the curved PM.

### (b) Curvature sensing: proteins with bilayer insertion motifs

In parallel, amphipathic helix motifs are another type of curvature-sensing domain (figure 2*c*). These helices might be structured in the soluble form of the protein, or be disordered domains folding upon interaction with the lipid bilayer. As has been discovered for ARFGAP1 (ADP-ribosylation factor GTPase-activating protein 1) [111], these helices screen lipid packing defects, where they can easily be buried. Curved areas of the bilayer generate more transient defects than planar regions, giving these helices curvature-sensing capabilities [112]. The helix of ARFGAP1 has given the name to a family of motifs, ALPS (amphipathic lipid packing sensors), but the mechanisms used by ALPS have since then been expanded to a wide range of amphipathic or hydrophobic motifs [113]. Alpha-synuclein and proteins with hydrophobic anchors (e.g. lipidated N-terminal domains) also sense lipid packing defects [114,115], and some reports suggest that many proteins containing such motifs have curvature-sensing properties [63,115]. However, there might be rules restricting curvature-sensing capabilities to some hydrophobic motifs with specific properties [112], or setting the sensed curvature size range [114]. N-BAR proteins also possess ALPS motifs, opening the debate about which motifs in the BAR superfamily of proteins are in fact sensing PM curvature [116]. Several studies found that ALPS motifs, rather than the BAR domains [117], were responsible for curvature sensing in AMPH and some F- and I-BAR proteins [118–120]. Some studies therefore claim that the sensing motif could be the ALPS motif only, with the driving force for its interaction with the PM being the density of defects rather than affinity [120]. How F-BARs or I-BARs that do not contain ALPS motifs would sense curvature is unclear, but crowding effects [121] or effects from the surrounding protein backbone [122] or the large disordered domains found in many of these proteins [123,124] could be involved. Most probably, the curvature sensing event is a cooperative process to which each of these domains contributes [125].

### (c) Sensing versus inducing curvature

If present at sufficiently high concentrations (as is often the case in assays *in vitro* or in overexpression conditions), CSPs not only sense, but also induce curvature [112,116,126]. Whether sensing and inducing are always mediated by the same physical mechanisms remains unclear, but theoretical models [80] suggest that curvature sensing and generation are two manifestations of a fundamental coupling between the bilayer free energy, the chemical potential of proteins and curvature. According to this view, sensing and generation would occur concomitantly in general and can only be uncoupled in situations such as dilute or highly crowded protein coverage or fixed membrane shape. Supporting this suggestion, AMPH has been shown to sense and generate curvature [126]. Accordingly, the curvature-inducing effect, analysed mainly via vesicle tubulation assays of GUVs *in vitro* [108], is often considered as proof that the proteins are also sensors [80]. In cells, CSPs organize and remodel the PM [127], and most studies focus on how these proteins actively induce curvature to optimize cellular functions such as endocytosis [128]. To decouple inducing from sensing effects, *in vitro* assays have been developed to study the sensing mechanisms only. These include assays to analyse the sensing of tense liposomes of different curvatures [119,120], lipid tether assays [126,129] and techniques such as the use of wavy lipid bilayers [130] or membrane-tubes extruded from a supported lipid bilayer [131]. It is also important to note that it is more challenging to study convex than concave curved proteins, although the former can be achieved by using lipid tethers [132]. Additionally, though many simulations have been developed to describe CSP bilayer shaping mechanisms [132,133], some also specifically describe sensing mechanisms [134].

In cells, CSPs may also trigger transduction to a biochemical signalling cascade. CSPs usually possess additional domains that recruit other partners, and convey a biochemical signal in the cell. BAR proteins localize to curved spots in the cellular PM [108], but this does not enable us to distinguish whether they sense curved PM domains or if they are recruited by another means (via lipid binding for example) and subsequently shape the PM, especially when overexpression of BAR proteins (known to induce PM tubule formation [127]) is used. The capacity of curved PM areas to recruit BAR proteins and induce mechanotransduction has mostly been found in cellular processes where PM curvature is pre-existing, and not generated in response to external forces. For instance, in CME (clathrin-mediated endocytosis), the endocytic bud (generated by CSPs themselves) possibly recruits other CSP participating in the endocytic event [135], although it is not clear when and whether curvature or other signals recruit these proteins [136]. During filipodia formation and retraction, invaginations created by PM tension release recruit the F-BAR protein FBP17 (formin-binding protein 17) [11]. Similarly, ArhGAP44 (Rho GTPase-activating protein 44), an N-BAR protein, colocalizes to nanoscale deformations in neurons, inhibiting filipodia formation/exploration [137]. The N-BAR protein PICK1 (protein interacting with C kinase-1) is recruited to nanovesicles (insulin-containing granules) because of their high curvature [138]. Other than BAR proteins, ARFGAP1, which contains an ALPS motif, is recruited to PM deformations induced by coat protein complex COPI [111].

### (d) Curvature sensing of deformations induced by extracellular forces

Other than responding to pre-existing PM curved areas, an exciting possibility is that curvature sensing is also important in the context of PM reshaping by extracellular stresses. For instance, CSPs recruit to mechanically induced curved structures in bacteria [139], and to nanocone-shaped PM invaginations created by substrate topography [78,79]. In the study by Zhao *et al.* [78], engineering substrate topography through nanopillars of different sizes and shapes led to recruitment of CSPs of different types, from N-BAR to I-BAR. Additionally, topography-induced CSP recruitment triggered mechanotransduction by enhancing endocytosis, via the recruitment of endocytic proteins such as clathrin and DNM2 (dynamin-2). Inspired by this method, a later study [140] used fluorescent N-BAR overexpression at low concentration to detect locations of cellular PM invaginations.

As suggested by these works, endocytosis seems to be a major cell response downstream of PM mechanosensing. Recently, the CLIC-GEEC (clathrin-independent carrier and GPI-AP enriched endosomal compartment) endocytic pathway has been demonstrated to respond to mechanical tension [62]. The pathway found is governed by VCL (vinculin) acting upstream of GBF1 (Golgi-specific brefeldin A-resistance guanine nucleotide exchange factor 1), but the actual molecular sensor of the change in tension remains unknown. Two BAR proteins involved in CLIC-GEEC endocytosis and acting downstream of GBF1/ARF1 (ADP-ribosylation factor 1), namely IRSP53 (insulin receptor substrate protein, also known as BAIAP2) and PICK1 [141], could potentially play a role. In different work, a burst in endocytosis mediated by the BAR protein GTPase regulator GRAF1 (also known as ARHGAP26, Rho GTPase-activating protein 26) was reported upon restoring medium tonicity after a harsh hypotonic treatment [105], but the driving factor of GRAF1 recruitment remained unclear. The treatment also led to formation of VLD and subsequent recruitment of GRAF1. However, whether the curvature of micrometre-sized VLDs matches the sensing capabilities of GRAF1 is unclear. Interestingly, endocytosis in both studies is mediated by accumulation of myristoylated ARF proteins. In those proteins and similarly to ALPS motifs, the myristoylated hydrophobic anchor could insert into the PM and sense curvature.

Unrelated to endocytosis, the F-BAR RhoGAP protein Spv1 (spermathecal physiology variant) also has an interesting mechanosensing role in egg fertilization in *Caenorhabditis elegans* [106]. In the relaxed spermatheca, Spv1 localizes to the apical PM, inhibiting Rho1/RhoA activity. The entry of oocytes into the spermatheca stretches spermatheca cells, probably unfolding and flattening Spv1-containing microvilli, and leading to Spv1 detachment from the PM. Rho1/RhoA activity is then promoted, inducing cell contraction and expulsion of the fertilized embryo to the uterus. In this study, it is not clear whether Spv1 is recruited to PM microvilli through curvature sensing or another mechanism, but it seems clear that tension increase and subsequent microvilli flattening lead to Spv1 detachment, triggering signalling [106]. Other than these examples, further curvature-sensing events and subsequent mechanochemical responses likely remain to be unravelled. For instance, the reservoirs we observed upon cellular destretch [31] might be regions for curvature-sensing-mediated mechanotransduction.

### (e) Sensing protein relocation: response to phase changes

Upon decreasing PM tension, PIs cluster in invaginated PM structures in yeast [58], possibly due to either lipid sorting or changes in lipid phases. PI clustering led to binding of the PH (pleckstrin homology) domain of TORC2 to the PI-enriched PM, and consequently drove TORC2 recruitment and inactivation (figure 2*d*). Further, the enrichment of lipid packing defects in curved L_o_ domains upon compression could affect the location of anchored proteins. For instance, N-RAS (GTPase NRas), which targets lipid packing defects, shows preference for L_d_ domains on flat bilayers, but for L_o_ domains in highly curved liposomes [65].

## 5. Theoretical modelling

Much of the current understanding of the PM as a mechanochemical transducer reviewed here is the result of close interaction between experiments and theory. Theoretical models and simulations provide a framework to rationalize and predict both (1) how mechanical stimuli affect the PM, and (2) the complex ensuing PM mechanochemistry. Regarding (1), free-standing membranes are well described by classical Helfrich-like models, which consider the membrane as a continuum surface whose stable configurations minimize bending energy. This bending energy depends on the curvature of the surface, and is subject to constraints such as fixed volume or area (see also box 3). The mechanics of membranes adhered to deformable or active networks, however, is richer and far less explored. Continuum models have shown how the interaction between the PM and the actin CSK controls PM tension in pressurized blebbing cells [37], in motile cells [142] or during localized membrane perturbations [50]. These works highlight how friction or heterogeneous attachment to the CSK leads to significant tension gradients. Continuum simulations have also established the mechanisms by which membranes confined to deformable and possibly poroelastic substrates cope with excess area or interstitial fluid [59,61]. The PM–CSK interaction described by these continuum models ultimately depends on an intimate and dynamical coupling over multiple length scales [143,144], which remains to be fully understood. Turning to membrane mechanochemistry, phase separation in model membranes has been successfully modelled using molecular dynamics (MD) [145] and continuum thermodynamic models, which have also described the coupling between shape and composition [146,147]. The coupling between phase behaviour and tension, however, remains controversial since theoretical models predict mixing upon tension increase [55], whereas experiments suggest this and the opposite behaviour [54,148]. Furthermore, theoretical models having focused on model membranes; their applicability to the more heterogeneous and dynamical PM is unclear.

Box 3. **Box 3. Theoretical definitions.**

(*a*) *Continuum models*

Continuum models treat the PM as a continuous surface rather than resolving individual lipid molecules. This results in a mean field description of the response of the PM.

(*b*) *Continuum Helfrich model*

This model treats the PM as a surface whose local area cannot be easily changed (inextensibility), which can shear in-plane without storing elastic energy because it is fluid, and which stores elastic energy when it is bent. Mathematically, this leads to an energy function that penalizes deviations between the local curvature of the surface and a spontaneous curvature encoding the bilayer asymmetry. Helfrich conceived of such a model in 1973 [192].

(*c*) *Flory–Huggins model*

This model describes the free energy of mixtures of fluids or gases. One can imagine a simplest mixture to be binary. If the mixture consists of repelling fluid particles, a low-energy state can be devised in which particles will segregate into distinct pure phases. At finite temperature, however, the mixture will have a tendency to maximize entropy, which favours a homogeneous mixture. In general, these two mechanisms will compete. Since the entropic response depends on the temperature, one can envision a critical temperature for repelling fluid mixtures beyond which the mixture would be homogeneous, while the phases are separated for temperatures below the critical temperature. Such a model was conceived by Flory and Huggins in the 1940s [160,161] to describe the behaviour of a mixture of polymers and has hence been used to predict the response of various kinds of mixtures. When applied to protein gases on fluid membranes, curvature modifies the energy required to place a protein molecule in a given membrane location (its chemical potential), which can lead to protein-rich curved domains in conditions where a planar membrane would remain homogeneously mixed.

Much of membrane mechanochemistry hinges on the interaction between membranes and proteins [132]. To capture the specificity of these interactions, all-atom MD simulations have identified molecular mechanisms behind curvature generation [149] and maintenance [150] by BAR domains, or the gating of mechanosensitive channels by membrane tension [151]. At the expense of molecular specificity, coarse-grained MD simulations [152] have been able to reach micrometre-sized domains during microseconds to understand curvature sensing and generation by large numbers of isotropic [153] or banana-shaped proteins [154], and the tension-dependence of such processes [107].

These approaches are complementary to hybrid continuum/discrete models, which treat the membrane as a continuum elastic surface but treat individual proteins as discrete objects. Such hybrid models have examined how PM tension and elasticity control mechanosensitive channels [155], or protein-mediated interactions between curving proteins of different shapes [156,157]. They have also shown that the collective behaviour of many channels [158] or curving proteins [157,159] is fundamentally multibody, highlighting a fundamental gap between models for individual proteins and mean-field models treating proteins as concentrations [80]. The latter models couple Helfrich-like bending energies, in which the spontaneous curvature of the membrane depends on the concentration of curving proteins, with mean-field models for the free energy of the protein gas. Such mean-field models (such as that by Flory [160] and Huggins [161]) account for the mixing entropy of the proteins in the membrane, and the self-interaction between proteins (see also box 3). Such thermodynamic continuum models provide a self-consistent description of curvature sensing, sorting [126] and generation, as well as its coupling with membrane tension [162]. Strikingly, despite the fact that these phenomena can be highly dynamical and concomitant, current theories have focused on equilibrium, or considered restricted dynamics with either fixed shape [163] or fixed protein coverage [164]. Looking forward, a fundamental challenge in the field is to connect models capturing protein–lipid specificity and membrane mechanochemistry at a mesoscale.

## 6. Concluding remarks

Though external mechanical forces play a crucial role in cell fate, the role of mechanochemical feedback mediated by the cellular PM remains largely unexplored. To further advance in this promising area, several aspects need to be considered. First, PM homeostasis is highly cell-dependent, and efforts need to be made to mimic physiological forces as closely as possible. Second, the respective effects of the PM and the underlying CSK need to be further clarified, as both can be sensitive to similar stimuli and can modify each other. For instance, a protein recruited to focal adhesions, Vinculin, was recently described as an early player in the endocytosis response triggered by PM compression due to stretch release [62]. Relatedly, changes in the PM order induced by cell shape have been associated with CSK reorganization [75]. In this regard, recently described molecular probes of PM tension [53] are promising tools to distinguish between these effects. Third, the respective roles and mechanisms of curvature sensing and inducing need to be further clarified. If all proteins bearing a hydrophobic anchor can sense curvature, this implies a future important broadening of the field, especially if a combined effect with phase transition occurs as with N-Ras recruitment to L_o_ domains in curved liposomes. Fourth, this review also highlights the interplay between tension and curvature sensing, and both seem to play a role in biochemical sensing mechanisms. In addition, external forces exerted on the PM trigger molecular rearrangements such as phase changes or lipid sorting, possibly affecting directly many signalling proteins [165]. Finally, many CSPs have been explored at the nanoscale, but how cells sense curvature at the microscale remains unclear [166]. Upon cellular compression, structures of very different sizes can be generated [31], and may induce very different curvature-sensing mechanisms. To conclude, mechanotransduction by the cellular PM is still an emerging field, which could even have implications in other cellular membranes [167], including the nuclear membrane [168,169]. Here, we explored the short-term mechanotransduction events involved. Interestingly, several of the molecular players discussed (BAR proteins [170–172], TORC2 [173], MGCs [174] Arf1 [175,176] and caveolae [177,178]) are involved in different cancer scenarios, potentially through altered mechanical responses. However, how this occurs, and how it is linked to long-term cellular response to mechanical signals, remains as an open question and is likely to be an exciting area of research.

## Data accessibility

This article does not contain any additional data.
